# Recent progress in the clinicopathological characteristics of alveolar soft part sarcoma

**DOI:** 10.3389/fmed.2025.1702870

**Published:** 2025-12-09

**Authors:** Nan Cong, Qi Shi, Qingyu Xu, Lin Zhang

**Affiliations:** 1 Department of Pathology, Mudanjiang Tumor Hospital, Mudanjiang, Heilongjiang, China; 2 Department of Pediatric Surgery, Hong Qi Hospital Affiliated to Mudanjiang Medical University, Mudanjiang, Heilongjiang, China; 3 School of Nursing, Mudanjiang Medical University, Mudanjiang, Heilongjiang, China; 4 The First Clinical Medical College of Mudanjiang Medical University, Mudanjiang, Heilongjiang, China

**Keywords:** alveolar soft part sarcoma, TFE3 gene fusion, immunohistochemistry, pathology, ASPS

## Abstract

Alveolar soft part sarcoma (ASPS) is a rare malignant soft tissue neoplasm characterized by distinctive histological features, indolent growth patterns and a propensity for metastasis. Recent advancements in molecular pathology and targeted therapies have significantly enhanced our understanding of its clinical and pathological characteristics. This paper systematically reviews the epidemiological profile, histopathological diagnostic criteria, molecular genetic mechanisms (notably the ASPSCR1-TFE3 gene fusion), immunophenotypic features, and differential diagnosis of ASPS. By integrating insights from multi-omics technologies, we explore the potential of personalized diagnostic and therapeutic strategies to improve disease comprehension and refine clinicopathological diagnostic standards, thereby guiding clinical management and enhancing patient outcomes.

## 1 Introduction

ASPS was first described and named by William M. Christopherson at the University of Washington in 1952 (1, 2), and was subsequently recognized by the World Health Organization (WHO) as a malignant neoplasm of uncertain histogenesis. ASPS accounts for approximately 0.5–1.0% of all soft tissue sarcomas (3). It predominantly affects adolescents and young adults (4). Clinically, ASPS typically presents as a slowly growing, painless masses; however, these tumors exhibit a high tendency to metastasize, particularly to the lungs and brain. Histologically, ASPS is characterized by epithelioid cells arranged in nests or acinar patterns, with cytoplasm rich in glycogen particles and an interstitium abundant in thin-walled blood vessels. Recent advances in molecular pathology have established a strong association between ASPS and the t(X;17)(p11;q25) chromosomal translocation, which results in the fusion of the ASPSCR1 and TFE3 genes (5). This discovery has opened new avenues for both diagnostic approaches and targeted therapies.

## 2 Methodology

This study aims to systematically review recent advances in the clinicopathological features of ASPS. To comprehensively gather relevant evidence, a systematic literature search strategy was developed. Databases including PubMed and China National Knowledge Infrastructure (CNKI) were searched using keywords such as “Alveolar Soft Part Sarcoma,” “ASPS,” “TFE3,” “ASPSCR1” and their combinations. The search covered records from the inception of each database up to September 2025. Inclusion criteria consisted of original studies, case reports, and systematic reviews reporting clinicopathological characteristics, molecular mechanisms, or treatment outcomes of ASPS, with publications limited to English and Chinese. Exclusion criteria included conference abstracts, unpublished full texts, articles irrelevant to the research focus, and duplicate publications. Although this article is a narrative review and does not strictly adhere to the full screening process of systematic reviews (such as PRISMA), the above systematic search and selection strategy aimed to minimize selection bias and ensure the representativeness and timeliness of the content reviewed. Ultimately, included literature was selected based on thematic relevance. Given the rarity of ASPS, most of the evidence cited in this review derives from retrospective analyses or small-sample studies, which may present certain limitations.

## 3 Epidemiological and clinical features

### 3.1 Epidemiological characteristics

**Figure 1:**
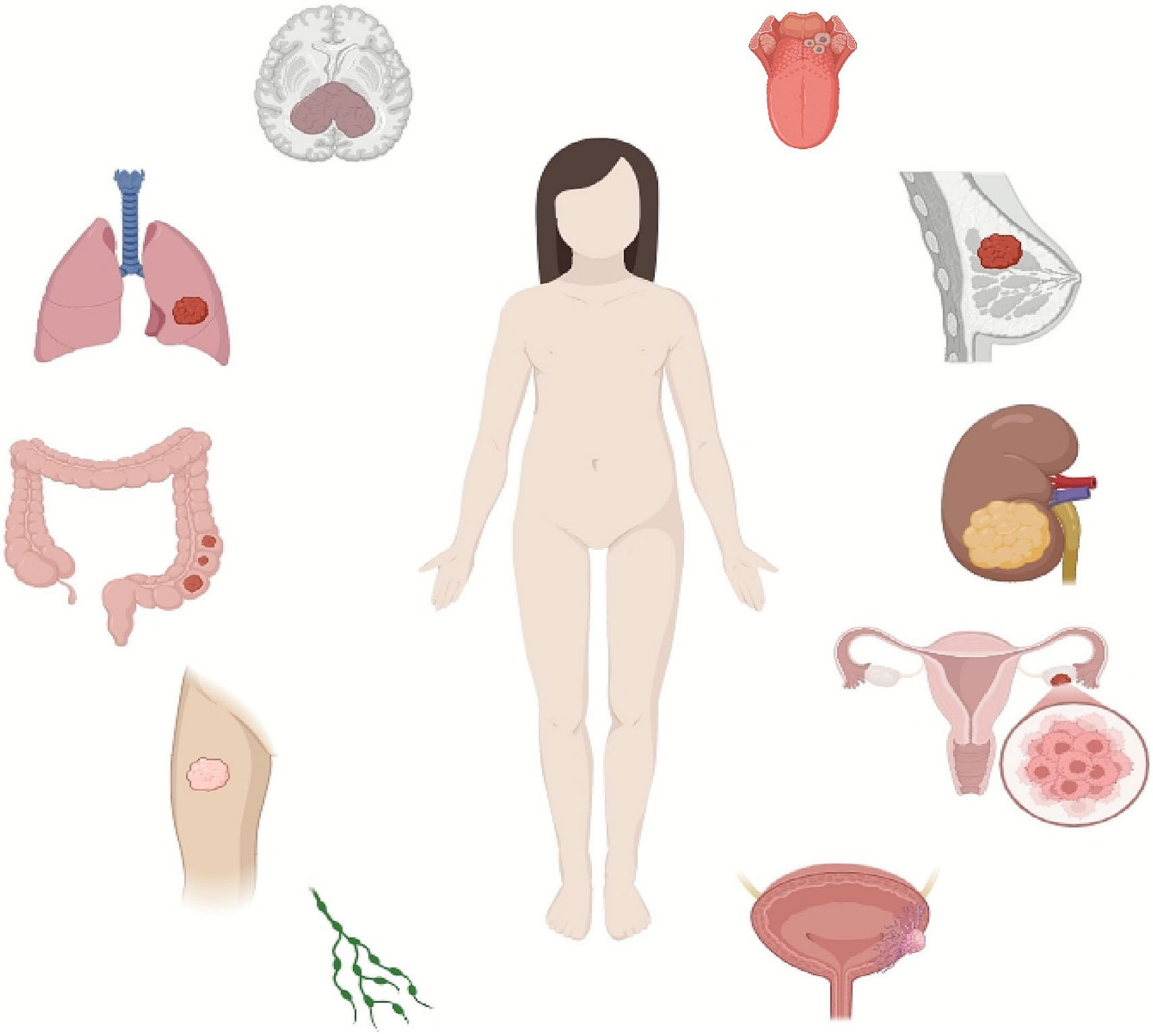
Common anatomical sites of ASPS primary tumors and metastasis. This diagram summarizes the predominant locations for ASPS development and spread. And highlights the higher incidence in limb deep soft tissues for adults and head/neck regions for children, along with frequent metastases to lungs, bones, and brain at diagnosis, underscoring the tumor’s aggressive nature (drawn by biorender.com).

The incidence and progression of ASPS are associated with patient age and gender. The highest incidence is observed in individuals aged 15–35 years (6), while it is less common in children and the elderly. Available data suggest a higher incidence in females than in males, with reported a male-to-female ratio of approximately 1:1.5 (7). No significant racial or regional differences in incidence have been identified. In adults, ASPS predominantly occurs in the deep soft tissues of the limbs, most commonly in the thigh (8), followed by the trunk (9), prostate, uterus (10), bladder (11), and breast (12). In pediatric and adolescent patients, ASPS is more frequently found in the head and neck region (13), particularly in the orbit and base of the tongue (14). A study by Genevois et al. (5) on children aged 0–18 years indicated that ASPS accounted for less than 1% of all soft tissue sarcomas, and approximately 42% of pediatric patients presented with metastasis at diagnosis. However, they also reported that the 5-year survival rate in children was about 95%, higher than that of adults (85%). ASPS exhibits slow growth, and early symptoms are often inconspicuous, leading to frequent delays in diagnosis and treatment. This disease has a high metastatic potential, and a significant proportion of patients have detectable metastases at initial diagnosis. Hematogenous metastasis is common. A comprehensive review by Tomohiro et al. (15) (study year up to 2022) reported metastasis frequencies at diagnosis as follows: lungs (approximately 70%), bones [30%, based on data from Xinbo et al. (16)], brain [15%, as cited from Shilpa et al. (17)], and lymph nodes [7%, from a case report by Xianmin et al. (18)] (Figure 1). The long-term prognosis for ASPS is poor, especially following distant metastasis. The overall mean survival time is approximately 7 years, A population-based analysis by Lieberman et al. (19) (covering data from 1923 to 1986) reported 2-year, 5-year, and 10-year survival rates of about 87, 62, and 43%, respectively, highlighting the poor long-term prognosis. Another study (20) found that the 5-year survival rate for localized ASPS was 86%, compared to 62% for metastatic disease. Conversely, an analysis of the SEER database by Haotong et al. (21) (study period 1973–2014, *n* = population-based cohort) reported a lower 5-year overall survival rate of 56%, indicating potential variations based on study population and time period.

### 3.2 Clinical manifestations

Systemic symptoms are typically absent in the early stages of ASPS. However, as the disease progresses to intermediate or advanced stages, patients may experience general fatigue, weight loss, and malnutrition. Most local symptoms manifest as painless masses (22), occasionally accompanied by local compression effects that can lead to peripheral nerve compression, vascular compression, or tissue compression, resulting in conditions such as nerve paralysis, limited limb movement, or movement disorders causing pain and discomfort. Given its propensity to metastasize to the lungs, brain, and bones (15, 16, 17) ASPS may present with symptoms such as coughing, dyspnea, dizziness, headaches, neurological dysfunction, limb pain, or pathological fractures. Most patients are diagnosed during medical consultations prompted by physical examinations or the onset of symptoms in the intermediate or advanced stages of the disease.

### 3.3 Imaging findings

X-ray examinations of patients may reveal poorly defined soft tissue masses, typically without calcification. Adjacent bone tissue may exhibit compression erosion or periosteal reaction, although bone destruction is uncommon. Ultrasonography usually shows an uneven hypoechoic mass with margins that may be clear or blurred due to peripheral invasion (23). The vasculature within ASPS tumors is characterized by sinusoidal, dense, hyperplastic, and fused vessels, often accompanied by dilation and remodeling. Color Doppler ultrasound frequently demonstrates abundant blood flow signals, indicating a rich vascular supply (24, 25). CT scans reveal ill-defined soft tissue masses with heterogeneous density. Post-contrast scans typically show heterogeneous enhancement, indicative of hypervascularity (26). CT also reveal compression erosion or periosteal reaction in adjacent bone tissue. MRI examinations show equal or slightly lower signal intensity on T1-weighted images, with internal signal heterogeneity due to hemorrhage or necrosis (27). On T2-weighted images, the mass exhibits high signal intensity with heterogeneous internal signals, revealing areas of hemorrhage or necrosis (28). Contrast-enhanced scans display heterogeneous tumor enhancement, sometimes appearing as a “honeycomb” pattern (29, 30). MRI clearly delineates the relationship between the tumor and surrounding muscles, blood vessels, and nerves. Positron emission tomography-computed tomography (PET-CT) imaging indicates high metabolic activity within the tumor, with a high maximum standardized uptake value (SUVmax) (31), aiding in the evaluation of malignancy and metastasis. Angiography demonstrates abundant tumor blood supply, with prominent tumor vessels and early venous filling. Due to the slow progression and lack of obvious early symptoms, patients often seek medical attention years after symptom onset or following incidental findings during physical examinations, leading to delayed diagnosis and treatment that can adversely affect prognosis.

## 4 Pathological features

### 4.1 General morphology

ASPS tumors vary in size, with the largest cross-sectional diameter on axial images ranging from a few centimeters to >10 cm (32). The gross appearance is often lobulated or nodular, with poorly defined margins and frequently an incomplete or pseudocapsule (33). Upon sectioning, the tumor appears gray or grayish-yellow, with a soft to moderately firm consistency, and is often fragile, resembling jelly or fish flesh. The tumor is highly vascularized, frequently accompanied by hemorrhage, necrosis, or cystic changes (34), which can be exacerbated by trauma to the tumor site.

### 4.2 Histological features

ASPS is histologically graded into three levels: low-grade (I), intermediate-grade (II), and high-grade (III), based on the degree of tumor cell differentiation. Specifically, grade I tumors exhibit low malignant potential, whereas grades II and III are associated with higher malignancy (6). Due to their frequently asymptomatic nature in early stages, many cases are diagnosed at an advanced stage, corresponding to high-grade malignancy.

**Figure 2:**
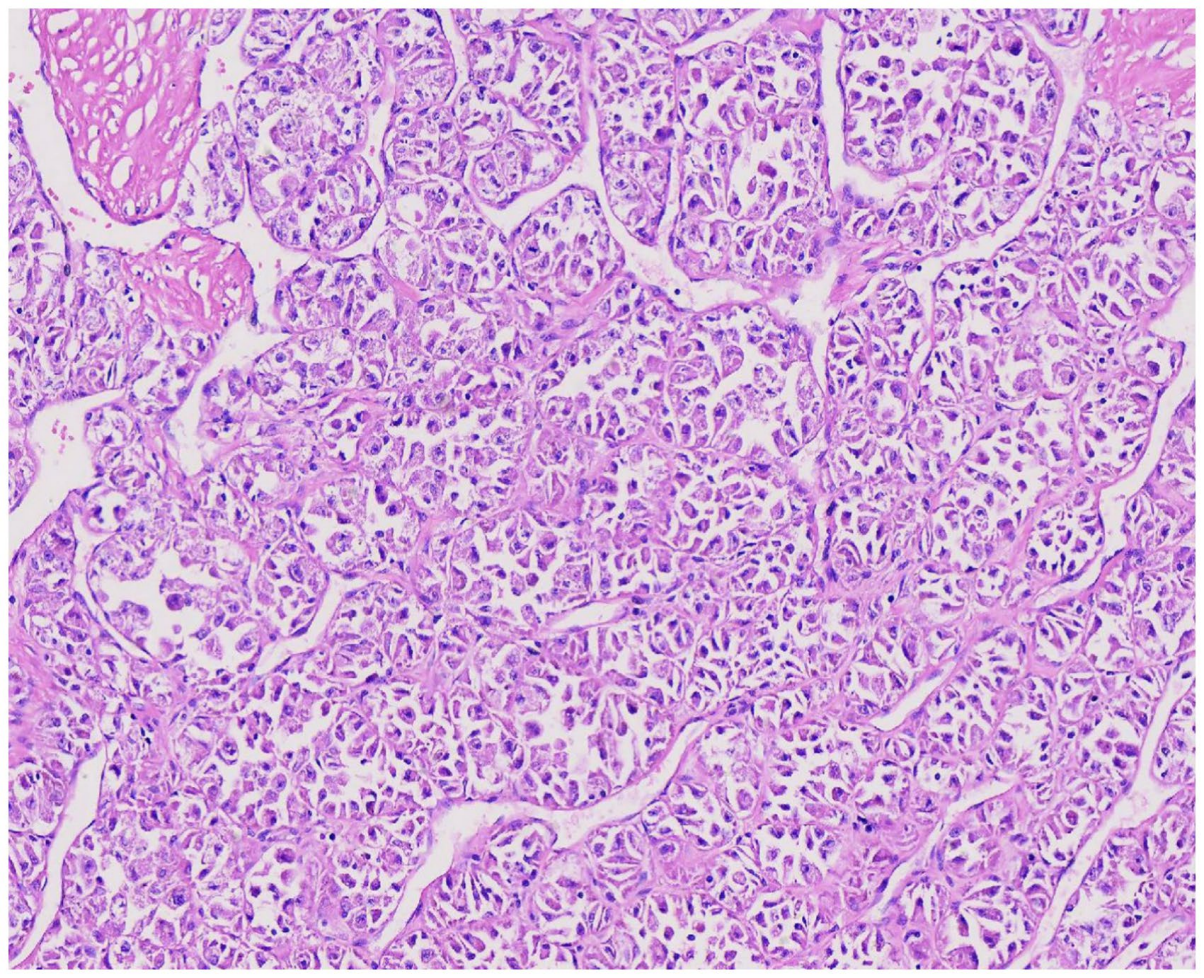
Under high magnification, tumor cells show classic features: large polygonal shapes with abundant eosinophilic cytoplasm, round/oval nuclei with prominent nucleoli, and a distinctive nested (alveolar) architecture separated by fibrovascular septa (H-E) (magnification factor 10 × 10).

A hallmark histological feature of ASPS is considered to be the arrangement of tumor cells into alveolar or nest-like structures, which may contain degenerative cells and pseudoglandular spaces when observed under low magnification (35). These nests are separated by thin fibrovascular septa, creating an “organoid” appearance (36). A rare histological pattern, observed in cases such as lingual tumors, results from necrosis within individual nests, creating a central clearing with tumor cells peripherally located, enhancing the alveolar appearance (37). In some pediatric cases, the tumor exhibits a diffusely solid growth pattern. At higher magnifications, tumor cells appear large and polygonal or round in shape, with abundant cytoplasm that may contain eosinophilic granules or clear vacuoles, and have distinct cell borders (Figure 2). The nuclei show mild pleomorphism, are round to oval, and are either centrally or eccentrically located with prominent nucleoli, fine chromatin, and an increased nuclear-to-cytoplasmic ratio. Mitotic figures are infrequent, although occasional cells display significant pleomorphism. Intravascular tumor emboli can be observed in peritumoral veins. The stroma contains abundant thin-walled vessels and a sinusoidal vascular network surrounding the tumor cell nests. Hemorrhage, necrosis, or fibrocystic changes may also be present in the stromal component.

Chuaychob et al. (38) developed a microfluidic co-culture chip using a mouse model to simulate the angiogenic microenvironment of ASPS and the intravascular cellular disorder preceding tumor metastasis. This system incorporated mouse ASPS cells, pericytes (PCs), and endothelial cells (ECs). Their results indicated that the proteins Rab27a and Sytl2 may play crucial roles in transporting bioactive signals to PCs and ECs during ASPS angiogenesis. With advances in imaging technology, Meng et al. (39, 40) employed apparent diffusion coefficient (ADC) histogram analysis to better capture tumor heterogeneity, thereby improving the prediction of pathological grading and supporting more accurate pathological diagnosis.

### 4.3 Immunohistochemical characteristics

**Figure 3:**
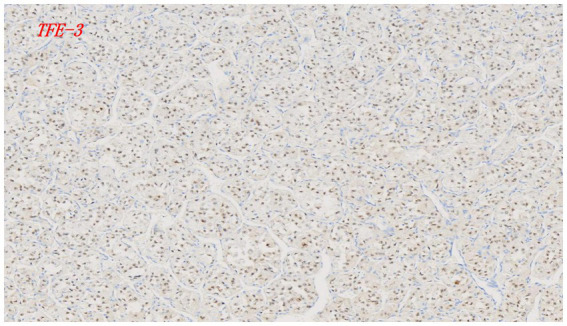
IHC staining of ASPS showed that the ASPCR1-TFE3 gene was fused and located in the cell nucleus, causing abnormal activation of TFE3 and showing positivity (magnification factor 10 × 10).

Nuclear overexpression of TFE3 is a sensitive and relatively specific feature of ASPS. Agaimy et al. (41) performed targeted RNA sequencing and fluorescence *in situ* hybridization (FISH) on 16 patients, successfully detecting TFE3 fusion in 14 cases (88%). This confirms the critical role of TFE3 immunohistochemical staining in diagnosing ASPS, with early studies reporting both sensitivity and specificity exceeding 95% (42, 43) (Figure 3). However, it is important to note that the diagnostic performance of TFE3 IHC can vary. Comparative studies have highlighted significant inter-laboratory variability, which may be influenced by factors such as the use of different antibody clones (e.g., polyclonal vs. monoclonal), staining protocols, and interpretation criteria. For instance, a comparative study by Sharain et al. (44) (sample size *n* = 125) indicated variability in TFE3 immunohistochemical (IHC) results depending on experimental reagents and antibody clones. The study reported suboptimal sensitivity and specificity in practice, suggesting that TFE3 IHC should play a minor role, if any, in diagnosing TFE3-rearranged tumors, and recommending FISH as the preferred method.

Vascular endothelial cells frequently express CD34 and vimentin, while the Ki-67 labeling index typically ranges from 10 to 20%. Although CD68 is commonly expressed in ASPS tissues, it lacks specificity as a diagnostic marker, as it is also expressed in various other cell types such as macrophages and monocytes. Some cases may exhibit focal weak positivity for myogenic markers such as desmin, MyoD1, and smooth muscle actin (SMA). Desmin typically shows focal weak positivity (4), whereas MyoD1 demonstrates cytoplasmic positivity in 60–70% of cases. Neurogenic markers like S-100 and neuron-specific enolase (NSE) can be present in some instances but lack specificity due to generally low expression levels. Melanocytic markers including HMB-45 and Melan-A are consistently negative in ASPS, which aids in differentiation from clear cell sarcoma (45). In addition to TFE3, cathepsin K also shows consistent diffuse positivity in ASPS, which is helpful for differential diagnosis against other tumors such as renal cell carcinoma, ASPCR1-TFE3 translocation renal cell carcinoma, adrenal cortex carcinoma, and paraganglioma (46, 47). Taylor et al. (48) show TRIM63 overexpression by RNA-ISH technology to be highly enriched within ASPS, with high levels of expression (H-score greater than 200) in 19/20 (95%) cases. Quantitative assessment of TRIM63 staining by RNA-ISH is potentially a helpful biomarker for ASPS with molecular TFE3 alterations. Accurate detection of ASPS biomarkers through IHC and *in situ* hybridization techniques is beneficial for the diagnosis, treatment, and prognostic intervention of diseases. Accurate molecular testing has the potential to shorten diagnostic time, reduce the risk of metastasis, and guide targeted therapy.

### 4.4 Ultrastructural characteristics

Pierre Masson initially identified and demonstrated the presence of cytoplasmic crystal structures in ASPS using Periodic Acid-Schiff (PAS) staining, confirming their resistance to diastase digestion (49). Electron microscopy studies have revealed that tumor cells contained distinctive rhomboid or rectangular crystalline inclusions within the cytoplasm, which are composed of periodically arranged parallel fibrils and show positive staining with PAS (50). These crystalline structures represent hallmark ultrastructural features of ASPS, although they are typically not visible in routine histopathological sections. The cytoplasm of tumor cells also contains abundant mitochondria, rough endoplasmic reticulum, and Golgi apparatus. Dysplastic desmosomal junctions were observed between tumor cells, while tight junctions and gap junctions were notably absent. Ultrastructural examination confirms the presence of these unique crystals, providing a valuable auxiliary diagnostic tool. However, due to the specialized equipment requirements, necessary technical expertise required, and the time-consuming and costly nature of ultrastructural analysis, this method is generally not the first-line diagnostic approach for ASPS.

### 4.5 Molecular genetic characteristics of ASPS

The hallmark molecular alteration in ASPS is the t(X;17)(p11;q25) chromosomal translocation, which results in the fusion of the ASPSCR1 gene on chromosome 17 with the TFE3 gene on the X chromosome. This genetic rearrangement leads to the formation of the ASPSCR1-TFE3 fusion gene, where the 5′ end of ASPSCR1 fuses with the 3′ end of TFE3, preserving the DNA-binding domain of TFE3 and part of the ASPSCR1 sequence. The presence of this fusion gene can be detected using FISH or reverse transcription polymerase chain reaction (RT-PCR), serving as a critical molecular marker for diagnosing ASPS (51). TFE3 is a transcription factor possessing intrinsic transcriptional activation properties. Following the chromosomal rearrangement, TFE3 expression becomes dysregulated, leading to its involvement in cell proliferation, metabolism, and immune modulation. Specifically, TFE3 upregulates genes associated with the transforming growth factor-beta (TGFβ) pathway, thereby altering the tumor microenvironment (52). The ASPSCR1-TFE3 fusion gene is believed to cause aberrant activation of TFE3, which is thought to promote angiogenesis, cell proliferation, differentiation, and inhibiting apoptosis, thereby potentially contributing to tumorigenesis and tumor progression (53). The t(X;17)(p11;q25) translocation not only results in the ASPSCR1-TFE3 fusion but also drives the abnormal activation of MET and PI3K/AKT signaling pathways (54), generating fusion proteins with oncogenic potential that promote tumor growth and metastasis.

Amir et al. (55) demonstrated co-dependence between ASPSCR1-TFE3 and Valosin-containing protein (VCP) in cancer cell proliferation and tumorigenesis in ASPS mouse models, suggesting VCP as a specific cofactor and potential novel therapeutic target. Nakamura et al. (56) developed an ASPS mouse model by introducing ASPSCR1-TFE3 into murine embryonic mesenchymal cells, elucidating a metastatic process involving peripheral cells enveloping blood vessels and tumor cell nests. Additionally, ASPSCR1-TFE3 has been shown to associate with active enhancers and super-enhancers, with the absence of ASPSCR1-TFE3 significantly reducing angiogenesis-related enhancers. In addition, morphological proteomics analysis provides insights into the biology of ASPS, namely that the ASPL-TFE3 fusion protein in ASPS binds to the mesenchymal-epithelial transition factor (MET) promoter, inducing the expression of cellular-mesenchymal epithelial transition factor (c-MET) tyrosine kinase. The discovery of phosphorylated c-MET indicates that it has been activated by its ligand, hepatocyte growth factor (HGF) (57, 58), further promoting the occurrence and development of ASPS.

## 5 Diagnosis and differential diagnosis

### 5.1 Diagnosis

Regarding diagnosis, in addition to conventional histological examination and immunohistochemical staining, several novel molecular diagnostic techniques have emerged in recent years. For instance, the detection of ASPSCR1-TFE3 fusion gene transcripts via FISH or RT-PCR has significantly enhanced the accuracy and specificity of diagnosing ASPS (44). These methods are crucial for differentiating ASPS from other soft tissue tumors that exhibit similar histological characteristics. However, the diagnosis of ASPS still faces significant challenges. Its rarity means many pathologists lack sufficient experience with it, and its histological similarity to various tumors (such as paraganglioma and metastatic renal cell carcinoma) easily leads to misdiagnosis. While TFE3 immunohistochemical staining is an important ancillary tool, the study by Sharain et al. (44) (sample size *n* = 125, evidence level 3) points out that TFE3 staining has issues with false positives and false negatives, and its specificity and sensitivity vary between laboratories, highlighting the risks of relying solely on immunohistochemistry. To reconcile the conflicting data, the reported sensitivity of TFE3 IHC ranges from approximately 88% to over 97%, while specificity can vary widely (approximately 65 to 99.5%) depending on the study cohort and methodology (41, 44). Therefore, a definitive diagnosis must rely on molecular detection [e.g., FISH, RT-PCR, or next-generation sequencing (NGS)] of the ASPSCR1-TFE3 fusion gene to resolve diagnostic ambiguity. Molecular assays play a critical role in confirming the specific fusion type and differentiating ASPS from its morphological mimics. For instance, while PEComas can share some histological features and may rarely harbor TFE3 rearrangements (often with different fusion partners), and RCC variants can exhibit clear cells and vascularity, the presence of the definitive ASPSCR1-TFE3 fusion is pathognomonic for ASPS. Based on current evidence, a recommended diagnostic algorithm is proposed: (1) Initial screening with TFE3 IHC, acknowledging its utility as a sensitive but not entirely specific marker; (2) For cases showing positive or equivocal TFE3 IHC results, confirmation with a molecular method such as FISH or NGS is mandatory to establish the definitive diagnosis of ASPS. Nevertheless, molecular testing techniques are not universally available in all medical institutions, and testing may fail for tumor samples with significant necrosis. The research by Tan et al. (59) on tertiary lymphoid structures (TLS) as biomarkers (*n* = 28, evidence level 3) offers a new perspective for diagnosis, but its clinical application value still requires validation through larger, multi-center studies (evidence level 2 and above). In summary, the precise diagnosis of ASPS requires a comprehensive approach integrating clinical presentation, imaging, pathological morphology, immunophenotype, and molecular genetics.

### 5.2 Differential diagnosis

ASPS exhibits numerous clinical and pathological features with other diseases, making differential diagnosis crucial. Key entities to differentiate include hemangioma, paraganglioma, metastatic renal cell carcinoma, alveolar rhabdomyosarcoma, synovial sarcoma, clear cell sarcoma, and granulosa cell tumor. Specific differentiation criteria are outlined in Table 1.

**Table 1:** Differential diagnosis.

Disease	Key features	Diagnostic markers	References
Muscular or intermuscular vasculature	Color ultrasound: the blood vessels inside the tumor are slender and linear, with extremely low degree of confusion; T2WI: high signal intensity shadow, uniform internal signal, clear edges; CT: calcification, enhanced scan shows significant nodule enhancement.	CD31 (+), CD34 (+).	(60, 61)
Paraganglioma	The thin-walled sinusoidal blood vessels encircle the cells into organoid structures, resembling alveolar soft part sarcoma. Mostly occurring in carotid bifurcation, jugular bulb, middle ear, and abdominal autonomic nerve trunks.	S-100, synaptophysin, CgA (+), TFE3 (−); gene-free fusion.	(4, 62)
Metastatic renal cell carcinoma	Microscopic examination revealed a malignant neoplasm with polygonal cells, abundant eosinophilic cytoplasm, eccentric nuclei and prominent nucleoli.	PAX8, CAIX (+), TFE3 can be positive without ASPSCR1 fusion.	(63, 64, 65, 66)
Alveolar rhabdomyosarcoma	It is more common in children and adolescents, grows rapidly, often accompanied by pain, and presents as isointense on T1WI and mixed high signal intensity on T2WI on imaging, with a high risk of necrosis.	Myogenin (+), MyoD1 (+), desmin (+). PAX3-FOXO1 or PAX7-FOXO1 gene fusions.	(67, 68)
Synovial sarcoma	Rapid growth, usually without crystal inclusions, and negative FISH or RT-PCR detection of TFE3.	EMA/CK (+), CD99 (+) SS18-SSX fusion gene expression increased.	(69, 70)
Clear cell sarcoma	Melanocyte markers are expressed, but TFE3 is not usually expressed	HMB-45 (+), melan-A (+), TFE3 (−)	(43, 71)
Granulosa cell tumor	The tumor cells were closely arranged, with fibrous septa, in the shape of nests. PAS staining showed no rod-like crystals in the cytoplasm	S-100 (+), NSE (+)	(72)

## 6 Conclusion and prospect

### 6.1 Summary

ASPS is a rare malignant soft tissue tumor characterized by slow growth and high metastatic potential. Clinically, it often presents as a painless mass, predominantly affecting adolescents and young adults, with a slightly higher incidence in females. It most commonly occurs in the deep soft tissues of the limbs and frequently metastasizes to sites such as the lungs, brain, and bones. Although it may exhibit an indolent clinical course, advanced disease is associated with a poor prognosis. Retrospective analyses indicate that gender and metastatic status are independent prognostic factors in ASPS patients. Percutaneous core needle biopsy is a standard preoperative diagnostic procedure. However, it carries risks including tumor cell dissemination, hemorrhage, and infection. Moreover, due to tumor heterogeneity and potential sampling errors, the procedure may yield false-negative results. Currently, surgical resection remains the primary treatment for localized ASPS, and wide excision with adequate margins is considered effective in reducing postoperative recurrence and metastasis. However, its efficacy is limited in cases of metastatic ASPS. Conventional chemotherapy and radiotherapy are generally ineffective against this tumor, underscoring its distinct biological behavior and resistance to traditional therapeutic approaches. Although targeted therapy and immunotherapy offer promising avenues, current evidence of their success is largely based on case reports or small phase II clinical trials, lacking validation from large-scale phase III randomized controlled trials. For instance, Osaki et al. (73) employed the NCC-ASPS2-C1 cell line to screen a drug library and identified several compounds that inhibit ASPS cell proliferation, thereby providing a valuable resource for advancing research and developing novel treatment strategies for this challenging disease. Immunohistochemically, ASPS cells often exhibit variable expression of markers such as Desmin, MyoD1, SMA, S-100, and NES. However, these markers generally lack sufficient sensitivity and specificity, limiting their diagnostic utility in clinical and pathological practice. Histologically, ASPS is characterized by a distinctive alveolar architecture, a rich vascular network, and intracytoplasmic PAS-positive crystalline inclusions. These features are key to distinguishing ASPS from other soft tissue tumors and help prevent misdiagnosis. The study of ASPS has evolved from morphological description to in-depth exploration of molecular mechanisms. Its molecular genetic profile is well-defined, with the characteristic alteration being a chromosomal translocation t(X;17)(p11;q25), which results in the formation of an ASPSCR1–TFE3 fusion gene. The identification of this fusion gene has transformed diagnostic and therapeutic strategies for ASPS. Moreover, nuclear positivity for TFE3 in immunohistochemical staining has become a cornerstone of ASPS diagnosis.

### 6.2 Clinical recommendations

**Figure 4:**
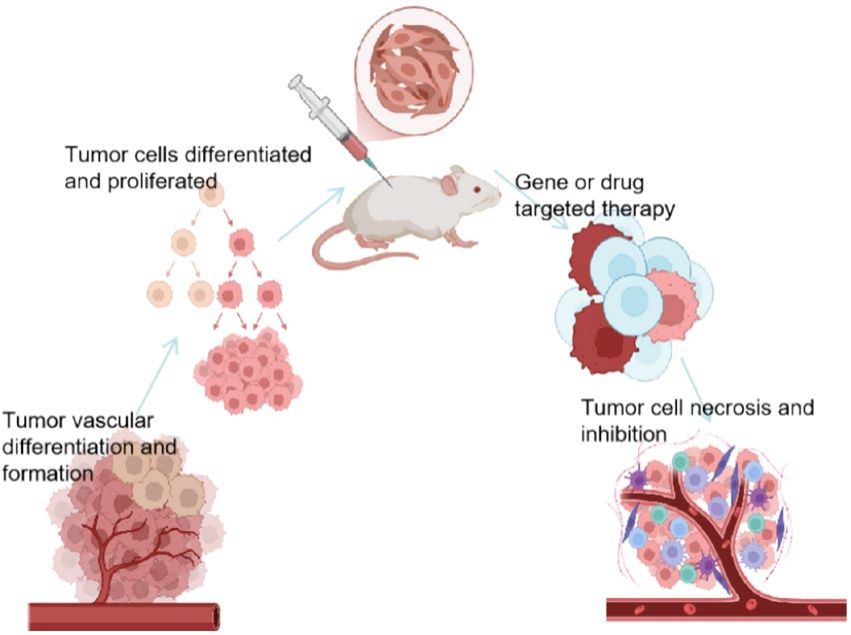
This schematic diagram illustrates the driving of tumor growth by activating carcinogenic pathways. And the inhibitory effect of targeted therapy on tumors was described (drawn by biorender.com).

In recent years, substantial progress has been made in developing treatment strategies that target the molecular features of ASPS. Accurate diagnosis, particularly the definitive molecular confirmation of ASPSCR1-TFE3 fusion, is paramount before initiating targeted therapies, as it ensures the correct patient population is selected, especially given the overlap in TFE3 positivity with other tumor types. A critical synthesis of the available evidence reveals that while several targeted and immunotherapeutic approaches show promise, the current body of evidence is largely derived from retrospective analyses and small phase II trials, with a notable lack of large-scale randomized controlled data. Given that the characteristic ASPSCR1-TFE3 fusion gene drives aberrant activation of signaling pathways such as MET and vascular endothelial growth factor (VEGF), tyrosine kinase inhibitors (TKIs) targeting these pathways, including anlotinib and cabozantinib, have been applied in the management of ASPS. A Phase II clinical trial (n = 28, evidence level 3) conducted by Tan et al. (59) investigated the efficacy of anlotinib combined with the PD-L1 inhibitor TQB2450 in patients with advanced ASPS. This regimen demonstrated a high objective response rate (ORR), providing encouraging preliminary evidence for the application of targeted-immunotherapy combination in this field (Figure 4). However, it must be noted that the current evidence is derived solely from a single-arm Phase II study, which has a limited sample size and lacks a control group comparing it to standard treatments. The long-term benefits, optimal treatment duration, and the combined toxicity profile of this regimen still require further validation in larger-scale Phase III randomized controlled trials (evidence level 1).

Furthermore, based on the hypothesis that the TFE3 fusion protein may serve as a neoantigen, adoptive cellular immunotherapies, such as T-cell receptor-engineered T cells (TCR-T) or chimeric antigen receptor T cells (CAR-T), are in the early stages of exploration. However, current research is confined to preclinical models or very limited case reports (evidence level 4). Significant challenges remain regarding their feasibility, the identification of suitable targets, and safety profiles. Despite these advancements, effective treatment options for patients with metastatic disease remain limited. The primary challenges involve overcoming therapy resistance, validating predictive biomarkers, and optimizing combination strategies to improve outcomes. Currently, the evidence base supporting the majority of treatment recommendations is weak, primarily relying on retrospective analyses and small Phase II studies (generally evidence level 3). There is a pressing need for higher-level evidence-based medical data to guide clinical practice.

### 6.3 Research directions

Future research should systematically employ spatial transcriptomics to delineate the transcriptional activity heterogeneity of ASPSCR1-TFE3 fusion gene transcriptional activity across different tumor regions. Integrated with single-cell RNA sequencing (scRNA-seq), this approach can delineate the specific gene expression profiles of distinct cancer cell subpopulations, immune cells, and stromal cells within the ASPS tumor microenvironment, thereby identifying key cellular subpopulations associated with metastasis and immune evasion. Concurrently, NGS of patient tissue or liquid biopsy samples can be used to screen for driver gene mutations and explore their correlations with clinicopathological features. Methodologically, multi-region sampling of surgical specimens from ASPS patients can be performed, combining scRNA-seq, whole-exome sequencing (WES), and multiplex IHC to quantitatively analyze intra-tumoral heterogeneity. Dynamic changes in circulating tumor DNA (ctDNA) should be monitored using droplet digital PCR (ddPCR) or NGS to evaluate treatment response and the emergence of resistance mutations. Furthermore, increased efforts in animal experimentation, particularly focusing on generating genetically engineered mouse models to observe spontaneous tumorigenesis, are warranted. Applying the CRISPR/dCas9 system *in vitro* to knock down or activate downstream TFE3 target genes (e.g., MITF family genes) can validate their roles in cell proliferation, apoptosis, and angiogenesis, providing an experimental foundation for clinical strategies. Concurrently, research should aim to identify molecular biomarkers correlated with ASPS prognosis to achieve early, precise diagnosis and effective disease monitoring, ultimately improving early detection rates. Multi-center clinical trials are essential to optimize combination targeted-immunotherapy regimens. This necessitates strengthened multidisciplinary collaboration among clinical and pathological specialties, oncology, radiology, and molecular biology to advance the precision diagnosis and treatment of ASPS. Establishing an international ASPS registry to aggregate comprehensive clinical and molecular data will foster extensive and in-depth research, enhancing global communication and cooperation. These efforts are anticipated to better guide individualized therapy and ultimately improve patient survival and outcomes.
